# A high-throughput study on endothelial cell adhesion and growth mediated by adsorbed serum protein *via* signaling pathway PCR array

**DOI:** 10.1093/rb/rbx030

**Published:** 2017-12-13

**Authors:** Xiaoying Lü, Yayun Qu, Ying Hong, Yan Huang, Yiwen Zhang, Dayun Yang, Fudan Zhang, Tingfei Xi, Deyuan Zhang

**Affiliations:** 1 State Key Laboratory of Bioelectronics, School of Biological Science and Medical Engineering, Southeast University, Nanjing 210096, P. R. China;; 2 Nanjing Drum Tower Hospital, Nanjing 210008, P.R. China;; 3 SQ Medical Device Co., Ltd., Nanjing 210008, P.R. China;; 4 Shenzhen Research Institute, Peking University, Shenzhen 518055, P.R. China and; 5 R&D Center of Lifetech Scientific (Shenzhen) Co., Ltd, Shenzhen 518057, P.R. China

**Keywords:** biomaterials, protein adsorption and cell adhesion, signaling pathway PCR array, biological signaling pathway

## Abstract

The purpose of this paper is to utilize the signaling pathway polymerase chain reaction (PCR) arrays to investigate the activation of two important biological signaling pathways in endothelial cell adhesion and growth mediated by adsorbed serum protein on the surface of bare and titanium nitride (TiN)-coated nickel titanium (NiTi) alloys. First, the endothelial cells were cultured on the bare and TiN-coated NiTi alloys and chitosan films as control for 4 h and 24 h, respectively. Then, the total RNA of the cells was collected and the PCR arrays were performed. After that, the differentially expressed genes in the transforming growth factor beta (TGF-β) signaling pathway and the regulation of actin cytoskeleton pathway were screened out; and the further bioinformatics analyses were performed. The results showed that both TGF-β signaling pathway and regulation of actin cytoskeleton pathway were activated in the cells after 4 h and 24 h culturing on the surface of bare and TiN-coated NiTi alloys compared to the chitosan group. The activated TGF-β signaling pathway promoted cell adhesion; the activated regulation of actin cytoskeleton pathway promoted cell adhesion, spreading, growth and motility. In addition, the activation of both pathways was much stronger in the cells cultured for 24 h versus 4 h, which indicated that cell adhesion and growth became more favorable with longer time on the surface of two NiTi alloy materials.

## Introduction

The developments of high-throughput biomics technologies provide powerful means to comprehensively understand the molecular mechanisms of how biomaterials impact organism from an overall genome/protein level. Bioinformatics analysis, an indispensable part of nowadays biomics research, extracts and interprets the biological significance of biomics data using some common techniques such as cluster analysis [1, 2], functional classification analysis [1–3], pathway analysis [1–2, 4–8], etc. A biological pathway is a series of actions among molecules in a cell that leads to a certain product or a change in the cell. Such a pathway can trigger the assembly of new molecules and turn genes on and off, or spur a cell to move. Pathway analysis is essential in the advanced biomics research, as it not only identifies affected pathways and the information of the involved genes, proteins, and other molecules, but also describes their molecular interactions and further pinpoints the affected steps within the pathways [9, 10].

Biological pathways have many classifications, and the most well-known pathways are metabolism pathways, gene-regulation pathways and signal transmission pathways (i.e. signaling pathways). Biological signaling pathways are an important mechanism to maintain biological viability. A signaling pathway transmits a physical or chemical signal from a cell's exterior to its interior by receptors and then by specialized proteins that trigger a cascade of biochemical events along the signaling pathway [10–12], to maintain biological processes. A biological signaling pathway contains a great amount of genes and the information of their interactions, which are important subjects of bioinformatics analysis and of great values for digging the biological significance of genes.

Besides using bioinformatics analysis of biomics data to identify important signaling pathways, they can be further filtered according to the effects of biomaterials corresponding to specific cell functions based on the cytology experiments and then be verified [13–17]. There are four major methods to study the signaling pathways. The first method is to inhibit the activity of a key protein within the pathway via competitive or noncompetitive binding of an antagonist, therefore revealing the function of the protein and the pathway [13]. The second method is to study a key protein and the function of its pathway by using an agonist to enhance the expression of the target protein [14]. The third method is to examine the expressions of a subset of the genes within a biological pathway by using a reverse transcription-polymerase chain reaction (RT-PCR) experiment [15]. And the fourth method is to use high-throughput signaling pathway PCR array to examine the expression profiles of the most important genes within the specific pathways and hence reveal the affected cellular functions [16, 17].

PCR array was developed as a class of functional classification arrays following the gene expression profile arrays. First proposed in 2002 based on PCR technique [18], it is able to detect at least 100 gene expression profiles simultaneously in one experiment. Compared with the RT-PCR, PCR array offers shorter experimental time and higher accuracy [19]. Currently, the signaling pathway PCR array is one of the widely-used PCR arrays which apply the microarray technology to biological pathways and targets the most important genes in a certain biological pathway. With PCR array, a simple, accurate, and reliable overview of multiple gene expression in a certain signaling pathway is provided simultaneously, and eliminates the time-consuming process of screening out genes related to the pathway one by one. So it is of great help in studying the function of the pathway in a fast and accurate way. However, there has no report in the literature of using the signaling pathway PCR array to validate the key pathways identified via bioinformatics analysis of biomics data.

**Figure 1.**
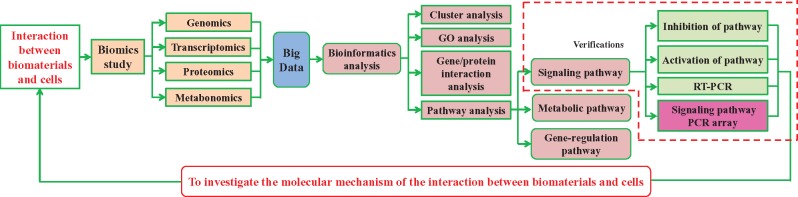
Our research strategy and technical roadmap to investigate the signaling pathways involved in the cellular interaction with biomaterials

Though the expressions of genes and proteins in cells can be detected by different biomics technologies with high-throughput and high sensitivity, false-positive or false-negative results in the experiments might be generated [20], thus bioinformatics analyses are required for further verification. The main verification methods include molecular biology and cell biology experiments, such as RNA interference, inhibition of signaling pathway, verification experiments at the cellular level [5–8, 21–23], etc. In order to study the mechanism of the interaction between biomaterials and cells, our group has adopted the ‘multiple biomics study + bioinformatics analysis + verification experiment’ research strategy (Fig. 1) for years. To verify the signaling pathway results obtained from bioinformatics analysis, we have investigated the function of the FAK-MEK-ERK pathway in PC12 cell differentiation induced by poly(L-Lactic Acid) (PLLA) aligned nanofibers by using pentapeptide GRGDS to interfere the integrin in PC12 cells. Both the expression of genes and proteins in the integrin-mediated FAK-MEK-ERK pathway and the PC12 cell differentiation were inhibited when integrins were blocked by GRGDS [22]. Moreover, by using specific signaling pathway inhibitors, it was found that the osteogenic-related markers of *Col*1 and *Gpnmb* were inhibited in bone marrow mesenchymal stem cells growing on natural hydroxyapatite (NHA) after blocking the ERK1/2 and the JNK mitogen-activated protein kinase (MAPK) pathways, therefore validating the significance of these two pathways in NHA-induced osteogenic differentiation [23].

NiTi alloy is widely used in the biomedical area, including the interventional treatment of cardiovascular disease due to their shape memory effect and superelastic effect. However, high nickel content in these alloys limits their applications. Thus, TiN coating has been used to improve the biocompatibility of NiTi alloy by reducing nickel release [6,24,25]. Biocompatibility researches on NiTi alloys and TiN-coated NiTi alloys were carried out at the molecular/cellular levels and at the whole animal level [6,26–28]. At the molecular level, our group previously studied the protein expression profiles of adsorbed serum proteins on the bare and TiN-coated NiTi alloys by using the label-free quantitative proteomics technology. Bioinformatics analysis showed that the adsorbed serum proteins on the two types of NiTi alloys could bind to the ligand proteins on the membrane of human umbilical vein endothelial cells (HUVECs) to activate intracellular signaling pathways and promote endothelial cell adhesion and growth. Especially, the TGF-β signaling pathway and the regulation of actin cytoskeleton pathway played important roles within this process. Then, the expressions of three TGF-β receptor genes (TGFBR1, TGFBR2, and TGFBR3) in TGF-β signaling pathway and three genes (VCL, PXN, FAK) in the regulation of actin cytoskeleton pathway were further studied by RT-PCR. The expression levels of these genes were significantly up-regulated, suggesting that the signaling pathway might be activated [6].

RT-PCR experiments can only study single or few gene expressions, whereas PCR array provides high-throughput information for the expressions of the most important genes as well as the activation of signaling pathway. Therefore, this paper aims to verify the proteomics results [6] by using PCR array and to further investigate the essential gene expressions in the TGF-β signaling pathway and the regulation of actin cytoskeleton pathway in HUVECs cultured on the bare and TiN-coated NiTi alloys. The differentially expressed genes in the pathways were identified and their influence on cell adhesion and growth was analysed to verify the significance of the two pathways in the cell adhesion and growth of HUVECs mediated by adsorbed serum proteins on the TiNi alloys. Furthermore, this study provides a novel approach to experimentally verify the signaling pathway obtained from bioinformatics analysis of biomics data, perfects our research strategy, and completes the technical roadmap for studying signaling pathways in the interaction between biomaterials and cells (Fig. 1).

Chitosan has good biocompatibility and has been widely used as a biomaterial [29]. Moreover, our previous research showed that the pure chitosan film had a certain inhibitory effect on the cell adhesion and proliferation of HUVECs, and the protein adsorption properties were obviously different among the chitosan, the bare and the TiN-coated NiTi alloy groups. Therefore, chitosan film was chosen as a control material in our previous research [6] and in this study.

## Materials and methods

### Sample preparation

The bare and TiN-coated NiTi alloy discs with 52 mm diameter and 1 mm thickness were supplied by Lifetech Scientific (Shenzhen) Co., Ltd. China. Vacuum filtered arc plasma deposition technique was used to form the 1-μm-thick TiN coating onto polished NiTi alloy. The NiTi alloy samples were cleaned by ultrasonication in acetone, ethanol, and ultrapure water for 10 min each before experiment [6].

The chitosan powder with 85% degree of deacetylation (Jinan Haidebei Marine Bioengineering Co, Ltd., China) was dissolved in the 2% (v/v) acetic acid to formulate the 2% (w/v) chitosan solution. The chitosan films were formed on the surface of 52-mm-diameter clean glass via spin coating (3000 rpm, 3 min). After evaporation in a convection oven at 45 °C for 12 h, the films were soaked into 1% (w/v) NaOH solution for 24 h and then in ultrapure water for 30 min, rinsed with ultrapure water three times, and finally dried at 45 °C for 12 h.

The bare and the TiN-coated alloys and the chitosan films were sterilized for 1 h each side by UV lamp before biological experiments.

### Cell isolation and culture

HUVECs were isolated from healthy human umbilical veins according to our previous method [6]. The HUVECs were cultured in medium 199 (Hyclone), supplemented with 20% (v/v) bovine serum (Hyclone), 30 μg/ml endothelial cell growth factor (ECGS) (Millipore), 1% (v/v) penicillin-streptomycin (Gibco) and 0.5% (v/v) heparin (medical grade), at 37 °C and under 5% CO_2_.

### Total RNA collection

The bare and TiN-coated NiTi alloys, as well as the chitosan films, were placed in tissue culture polystyrene dishes of 60 mm diameter (Corning, USA) separately. Passage 3-5 HUVECs were seeded on the surface of the NiTi alloys and the chitosan films with a cell density of 2.5 × 10^4^ cells/cm^2^ (surface area of 21.2 cm^2^, 5.3 × 10^5^ cells/sample) [6]. After incubated for 4 h and 24 h, respectively, the medium was removed, the samples were gently rinsed once with PBS and 1 ml Trizol buffer solution was then added. Total RNA were collected and stored at -80 °C.

### PCR array experiment and bioinformatics analysis

The PCR array experiments were carried out by Shanghai KangChen Bio-tech. The experimental procedure is summarized as follows: The total RNA in the cells was purified and then quantified using a Nanodrop ND-1000 UV/Vis spectrophotometer, and reverse-transcribed to cDNA. The PCR array experiments were performed using the human TGF-β/BMP Signaling Pathway Plus PCR Array and Cytoskeleton Regulators PCR Array (SABioscience, USA; both contained 89 genes). The gene expression values in the two pathways were calculated. Then the fold change of each gene was calculated by the gene expression value of the test sample (HUVECs on the surface of bare and TiN-coated NiTi alloys) dividing the gene expression value of the control sample (HUVECs on the surface of chitosan film). That is, the gene expression value of the control sample was normalized as 1. Next, the significantly differentially expressed genes were screened out: a gene was considered to be up-regulated when the fold-change value was more than 2 while down-regulated when the fold-change value was less than 0.5 (*P *<* *0.05). The significantly differentially expressed genes were further investigated by bioinformatics analysis, and their influence on endothelial cell adhesion and growth were discussed. The activations of the TGF-β signaling pathway and the regulation of actin cytoskeleton pathway, as well as their significance in mediating endothelial cell adhesion and growth on the surface of TiNi alloys were elucidated.

## Results and discussion

### Screening of significantly differentially expressed genes in PCR array

#### The experimental results of TGF-β/BMP signaling pathway PCR array

With the TGF-β/BMP Signaling Pathway Plus PCR Array, the gene expression profiles of HUVECs after 4 h and 24 h culturing on the bare and TiN-coated NiTi alloys and on the chitosan film were compared. The results showed that on the bare NiTi alloy, there were 30 genes with significantly differential expression at 4 h and 27 at 24 h; whereas on the TiN-coated NiTi alloy, there were 38 genes with significantly differential expression at 4 h and 31 at 24 h, respectively (see Supplementary Table S1–4). The detailed results of the up- and down-regulated genes associated with TGF-β signaling pathway are summarized in Tables 1 and 2.

**Table 1.** Genes in the TGF-β signaling pathway with significantly differential expression in the experimental groups (*P *<* *0.05)

No.	Gene symbol	Bare NiTi		TiN-coated NiTi	
		4h	24h	4h	24h
1	TGFB1	12.26	22.26	12.17	23.50
2	BMP6	6.75	6.42	4.53	5.05
3	NODAL	3.96	7.11	0.42	13.50
4	SMAD7	8.97	7.60	13.41	6.89
5	SMURF1	3.18	2.62	2.45	2.16
6	ACVR1	2.03	2.03	2.27	
7	MYC	2.55	3.44		2.76
8	BAMBI	2.30		3.08	2.46
9	TGFB3		2.41	0.32	2.50
10	SMAD6	4.60		4.72	
11	SMAD5			0.44	0.49
12	CHRD	7.27			
13	GDF6	2.11			
14	TGFBR2	2.09			
15	DCN	0.49			
16	NOG		3.04		
17	ACVR2A		0.45		
18	SMAD1			0.47	
19	TGFB2			0.13	
20	BMP4			0.12	
21	LEFTY1				9.24
22	THBS1				2.12
23	BMPR1A				0.48
24	BMPR1B				0.36

**Table 2.** The counts of differentially expressed genes and sum of expression values involved in the TGF-β signaling pathway in each experimental group

Group		Up-regulated genes		Down-regulated genes		Total number of differentially expressed genes
		Number	Sum of expression values	Number	Sum of expression values	
Bare NiTi	4h	12	58.07	1	0.49	13
	24h	9	56.93	1	0.45	10
TiN-coated NiTi	4h	7	42.63	6	1.9	13
	24h	10	70.18	3	1.33	13

**Figure 2.**
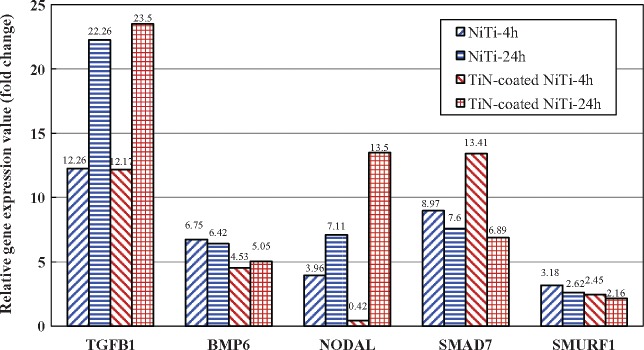
Five genes in the TGF-β signaling pathway that were differentially expressed on both the bare and the TiN-coated alloy groups

According to Tables 1 and 2, on the bare NiTi alloy, there were 13 and 10 genes with significantly differential expression involved in the TGF-β signaling pathway at 4 h and 24 h, respectively. On the TiN-coated NiTi alloy, there were 13 genes with significantly differential expression involved in this pathway at 4 h and 24 h. Overall, the number of the up-regulated genes were more than that of the down-regulated genes. And there were five genes that were differentially expressed on both the bare and the TiN-coated alloys (Fig. 2).

#### The experimental results of cytoskeleton regulators PCR array

**Figure 3.**
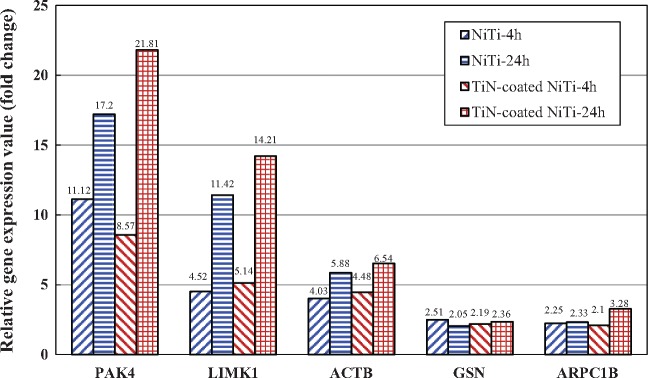
Five genes in the regulation of actin cytoskeleton pathway that were differentially expressed on both the bare and the TiN-coated alloy groups

The analysis results of cytoskeleton regulators PCR array showed that on the bare NiTi alloy, there were 30 and 24 genes with significantly differential expression at 4 h and 24 h, respectively. On the TiN-coated NiTi alloy, there were 29 genes with significantly differential expression in this pathway at both 4 h and 24 h (see Supplementary Table S5–8). The detailed results of the up- and down-regulated genes associated with the regulation of actin cytoskeleton pathway are summarized in Tables 3 and 4. As shown, on the bare NiTi alloy, there were 12 and 11 differentially expressed genes at 4 h and 24 h, respectively; while on the TiN-coated NiTi alloy, there 14 and 11 differentially expressed genes at 4 h and 24 h. Among these, there were five genes that were differentially expressed on both the bare and the TiN-coated alloys (Fig. 3). In addition, longer cell culture time resulted in more up-regulated genes and less down-regulated genes in this pathway for both NiTi alloys.

**Table 3.** Genes in the regulation of actin cytoskeleton pathway with significant differences in expression in the experimental groups (*P *<* *0.05)

No.	Gene symbol	Bare NiTi		TiN-coated NiTi	
		4h	24h	4h	24h
1	PAK4	11.12	17.20	8.57	21.81
2	LIMK1	4.52	11.42	5.14	14.21
3	ACTB	4.03	5.88	4.48	6.54
4	GSN	2.51	2.05	2.19	2.36
5	ARPC1B	2.25	2.33	2.10	3.28
6	WASL	2.50	3.50		2.26
7	DIAPH1	2.45	3.21		3.51
8	CFL1	2.02	2.66		3.05
9	MYLK	3.32		3.57	
10	ARPC2	0.46		0.40	
11	CDC42	0.46		0.30	
12	TIAM1		2.80		2.25
13	MYLK2	2.00			
14	WAS		4.04		
15	WASF1		2.25		
16	ARPC3			0.49	
17	ARPC5			0.37	
18	PIKFYVE			0.32	
19	PPP1R12A			0.31	
20	SSH2			0.29	
21	IQGAP2			0.26	
22	LIMK2				2.13
23	EZR				2.08

**Table 4.** The counts of differentially expressed genes and sum of expression values involved in regulation of actin cytoskeleton pathway

Group		Up-regulated genes		Down-regulated genes		Total number of differentially expressed genes
		Number	Sum of expression values	Number	Sum of expression values	
Bare NiTi	4h	10	36.74	2	0.92	12
	24h	11	57.34	0		11
TiN-coated NiTi	4h	6	26.06	8	2.74	14
	24h	11	63.50	0		11

### Bioinformatics analysis of significantly differentially expressed genes in TGF-β signaling pathway

TGF-β is a typical member of the ligand superfamily involve in regulating different aspects of intracellular stability, such as proliferation, differentiation, migration, and apoptosis. The TGF-β superfamily includes TGF-β subfamily, BMPs, activin, inhibin, etc., and they participate in TGF-β signal transduction [30, 31].

TGF-β signaling pathway incorporates several signaling pathways that share most components of a central signal transduction engine. The general scheme of signal transduction is: upon binding of a ligand, an activated plasma membrane receptor complex is formed, which passes on the signal towards the nucleus through a phosphorylated receptor SMAD (R-SMAD, including SMAD1, SMAD2, SMAD3, SMAD5, SMAD8). In the nucleus, the activated R-SMAD promotes transcription with a helper molecule SMAD4 [32, 33], and ultimately affects osteogenic differentiation, neuronal formation, apoptosis, cell cycle, etc. The signaling pathway includes many TGF-β superfamily ligands and several types of the R-SMADs. The R-SMAD: SMAD4 complex can interact with a large number of transcriptional co-activators/co-repressors to regulate positively or negatively effector genes [32, 34].

**Figure 4.**
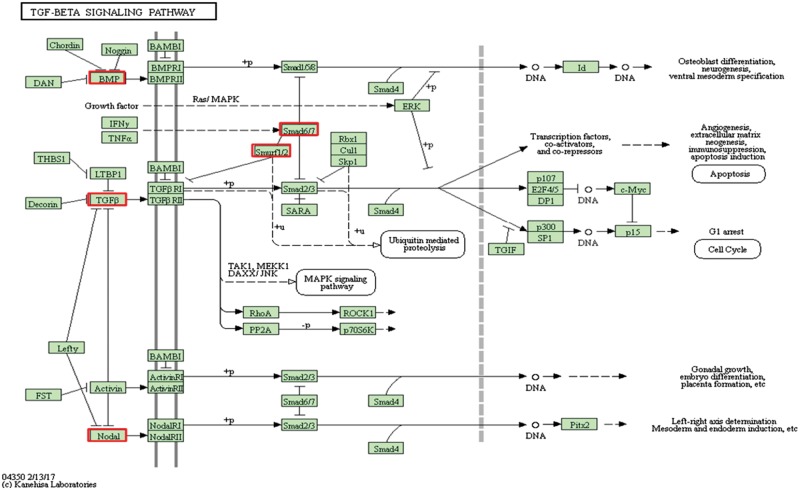
The TGF-β signaling pathway and the differentially expressed genes in both the bare and the TiN-coated NiTi alloy groups [35]

There were five genes that were differentially expressed on both the bare and the TiN-coated alloys (Fig. 2). Their locations and roles in TGF-β signaling pathway is shown in Fig. 4.

TGFB1 and BMP6 are members of the TGF-β ligand superfamily. TGFB1, a homodimer, binds to TGF-β receptor II (TGFBR2), inducing its dimerization. Binding of TGFB1 enables TGFB2 to form a stable complex with TGF-β receptor I homodimer (TGFBR1). TGFBR2 acts as a serine/threonine kinase, and phosphorylates serine and threonine residues within the short glycine-serine rich domain of TGFBR1 [32]. BMP6 binds to TGFB1, inhibiting SMAD2/3 activation, promoting SMAD1/5/8 activation, and thereby promoting the enhancement of TGFB1-mediated adhesion [36]. NODAL is a member of the TGF-β superfamily, and shares its type I and type II receptors and downstream signaling mediators (SMAD2/3 and SMAD4) with other TGF-β superfamily members such as TGF-β and activin [37]. TGF-β/activin/NODAL and BMP/GDP are two important subfamilies of TGF-β/SMAD signal. SMAD2 and SMAD3 function as the downstream transcription factors of TGF-β/activin/NODAL that bind to TGF-β1/-β3 receptors to be phosphorylated [38, 39].

SMAD7 is an inhibitory SMAD that acts as a negative regulator in the TGF-β signaling pathway. SMAD7 is induced by TGF-β, interacts with activated TGFBR1 and interferes the phosphorylation of the receptor-regulated SMADs [40]. SMURF1 is a target gene of SMAD that regulates TGF-β superfamily signaling through the dual mechanisms: (i) By interaction with and degradation of R-SMAD, SMURF1 negatively regulates BMP signaling. (ii) SMURF1 also interacts with SMAD7 and inhibits TGF-β signaling by receptor degradation [40, 41].

**Figure 5.**
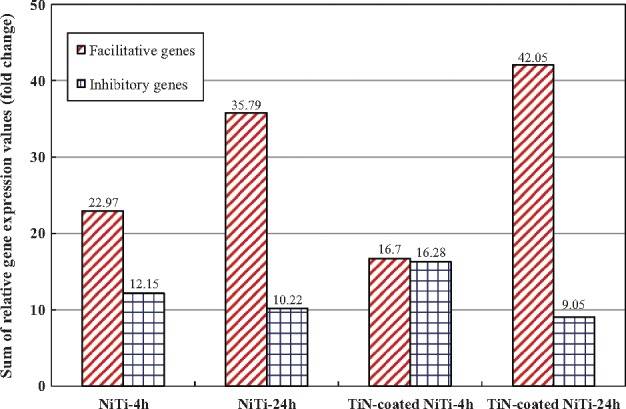
The gene expression values of facilitative genes and inhibitory genes in the TGF-β signaling pathways

According to the above analysis, among these five genes, three genes (TGFB1, BMP6 and NODAL) were facilitative and two were inhibitory (SMAD7 and SMURF1) to TGF-β signaling pathway when they were up-regulated. The expression patterns of these five genes and their effects on the pathway were summarized in Table 5; the sum of the expression values for the facilitative genes and inhibitory genes was further calculated and listed in Fig. 5, respectively. The results showed that, in the NiTi-4 h, the NiTi-24 h and the TiN-coated NiTi-24 h groups, all five genes were up-regulated as three (TGFB1, BMP6, and NODAL) of which were facilitative and two (SMAD7 and SMURF1) were inhibitory to the pathway. The number of the facilitative genes is more than the number of the inhibitory genes. While in the TiN-coated NiTi-4 h group, four genes other than NODAL were up-regulated. On the contrary that the up-regulation of NODAL activated the pathway, the down-regulation of NODAL inhibited the pathway. Thus, two genes (TGFB1 and BMP6) were facilitative and three (NODAL, SMAD7 and SMURF1) were inhibitory to TGF-β signaling pathway; the number of the facilitative genes was less than the number of the inhibitory genes. However, in all four experimental groups, the sum of expression values of the facilitative genes in each experimental group was higher than that of the inhibitory genes (Fig. 5), indicating that both NiTi alloys were favorable for activating the TGF-β signaling pathway in HUVECs. The results also showed that the sum of expression values of the facilitative genes increased with time while the sum of expression values of the inhibitory genes decreased (Fig. 5), indicating that the activation effect enhanced over time. In sum, compared to the chitosan film, TGF-β signaling pathway was activated in the HUVECs after 4 h and 24 h culturing on the bare and the TiN-coated NiTi alloys, resulting in promoting cell adhesion on the surface of the materials.

**Table 5.** Expression patterns of five differentially expressed genes and their effects in the bare and the TiN-coated NiTi alloy groups on TGF-β signaling pathway

No.	Gene symbol	Expression pattern and their effects on the pathway	Bare NiTi		TiN-coated NiTi	
			4h	24h	4h	24h
1	TGFB1	Expression pattern	↑	↑	↑	↑
		Effect on the pathway	+	+	+	+
2	BMP6	Expression pattern	↑	↑	↑	↑
		Effect on the pathway	+	+	+	+
3	NODAL	Expression pattern	↑	↑	↓	↑
		Effect on the pathway	+	+	—	+
4	SMAD7	Expression pattern	↑	↑	↑	↑
		Effect on the pathway	—	—	—	—
5	SMURF1	Expression pattern	↑	↑	↑	↑
		Effect on the pathway	—	—	—	—

↑: up-regulation, ↓: down-regulation, +: activating to the pathway, —: inhibitory to the pathway.

### Bioinformatics analysis of significantly differentially expressed genes in regulation of actin cytoskeleton pathway

Cytoskeleton as an important structure in the cell is a network made up of proteins; it maintains cell morphology, offers material transfer, facilitates cell movement, and supports cell differentiation. Cell signaling is a critical regulatory component of cytoskeletal involvement in cellular processes. Many related proteins such as the Rho family, MAPK, etc., interact with each other to affect cell morphology and functions through signal transduction associated with certain cytoskeleton regulatory pathways [42].

**Figure 6.**
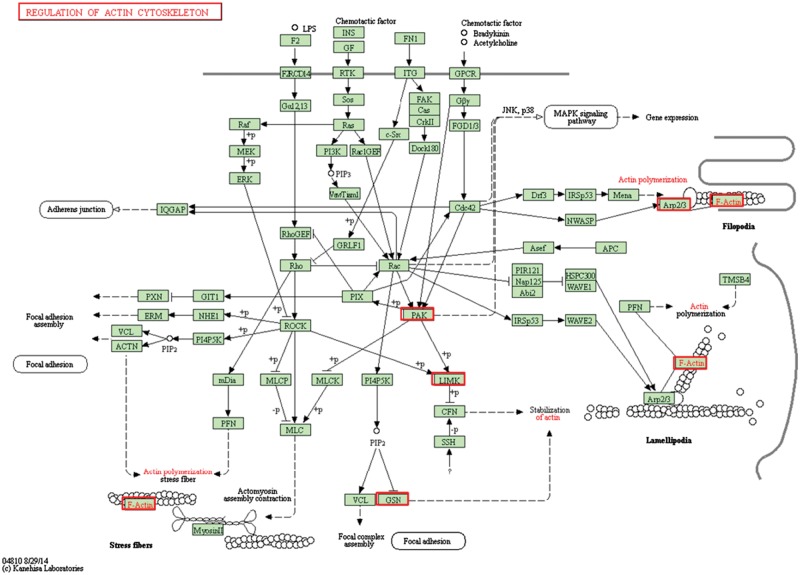
The egulation of actin cytoskeleton pathway and the differentially expressed genes on the bare and the TiN-coated NiTi alloys [35]

There were five genes that were differentially expressed on both the bare and the TiN-coated NiTi alloys (Fig. 2). Their locations and roles in the regulation of actin cytoskeleton pathway were shown in the Fig. 6.

The protein encoded by PAK4 is a member of the PAK family. PAK proteins are critical effectors link Rho GTPases to cytoskeleton reorganization and nuclear signaling. They serve as target for the small GTP binding protein Cdc42 and Rac, and have been implicated in a wide variety of biological activities. PAK4 plays a role in the reorganization of actin cytoskeleton as a mediator of filopodia formation [43]. PAK4 phosphorylates LIMK1 and LIMK1 phosphorylates cofilin. The phosphorylated cofilin is completely inactive, rendering the decrease of actin degradation, and thus resulting in stabilization of actin [44, 45]. ACTB is an F-actin and is important for the formation of focal adhesion. The increased expression of ACTB promotes the assembly of cytoskeletal stress fibers [46]. GSN is a widely distributed actin-binding protein that regulates cytoskeleton turnover [47]. GSN is a multifunctional regulator in the processes of cell motility, morphogenesis, and remodeling of actin cytoskeleton. Much of the researches on GSN have focused on its role in severing, capping, uncapping, and nucleating of actin fibers [48]. Studies have shown that the up-regulation of GSN promotes cell growth and motility [49]. ARPC1B encodes the p41 subunit of ARP2/3 complex, which is engaged in the assembly and structural maintenance of ARP2/3, and ARP2/3 complex regulates actin polymerization in the cells [50].

**Figure 7.**
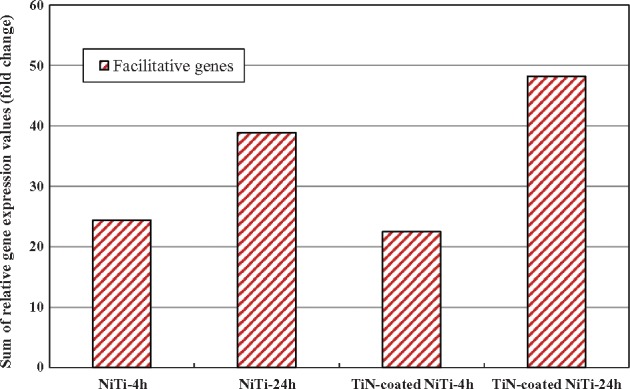
The gene expression values of facilitative genes in regulation of actin cytoskeleton pathway

According to the above analysis, these five genes all activated the regulation of actin cytoskeleton pathway and were all up-regulated in each experimental group (Table 3). The sum of the expression values is shown in Fig. 7, indicating that both NiTi alloys were favorable for activating the regulation of actin cytoskeleton pathway in the HUVCs. In addition, the sum of the gene expression values increased with time, indicating that the activation effect of both alloys enhanced over time. In summary, compared to the chitosan film, the regulation of actin cytoskeleton pathway was activated in the HUVECs after 4 h and 24 h culturing on the bare and the TiN-coated NiTi alloys, which promotes cytoskeleton stabilization, the assembly of cytoskeleton stress fibers, and cell growth and motility.

### Comparison analysis of PCR array results, proteomics results, and RT-PCR results

The results of the above-mentioned TGF-β signaling pathway and regulation of actin cytoskeleton pathway were compared with the previous proteomics results of the adsorbed proteins on the bare and the TiN-coated NiTi alloys [6], as shown in Table 6.

**Table 6.** The comparison analysis of PCR array results and previous proteomics and RT-PCR results

Material	Experiment name	Biological pathways	
		TGF-β signaling pathway	Regulation of actin cytoskeleton pathway
Bare NiTi alloy	Proteomics experiment of adsorbed proteins	A	A
	RT-PCR verification experiment	A	A
	PCR array	A	A
TiN-coated NiTi alloy	Proteomics experiment of adsorbed proteins	A	A
	RT-PCR verification experiment	A	A
	PCR array	A	A

A: Activation.

In our previous study, the expression levels of three genes (*TGFBR*1, *TGFBR*2, *TGFBR*3) in the TGF-β signaling pathway and three genes (*VCL*, *PXN*, *FAK*) in the regulation of actin cytoskeleton pathway have also been detected by using RT-PCR; these six genes were significantly (*P *<* *0.05) or highly significantly (*P *<* *0.01) expressed in the bare and the TiN-coated NiTi alloy groups compared to the chitosan group. This indicated that both alloys enhanced the activation of the TGF-β signaling pathway and the regulation of actin cytoskeleton pathway [6] (Table 5). Although the genes in a signaling pathway detected by the PCR array in this paper were not exactly the same of the genes detected by the previous RT-PCR experiment, both approaches have demonstrated the activation of these two pathways from a different perspective, and therefore have validated the proteomics results of bioinformatics analysis.

**Figure 8.**
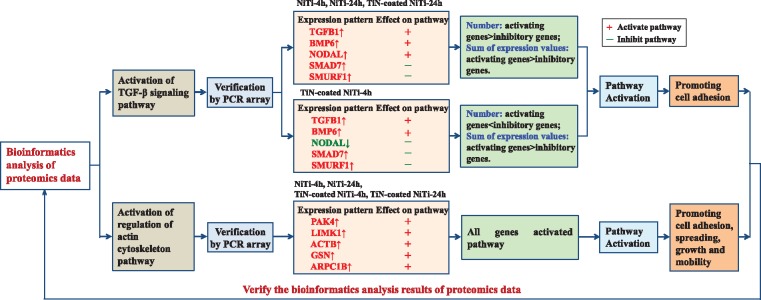
The roadmap to verify the bioinformatics analysis results of proteomics data by the PCR array

Since the RT-PCR experiment is only able to detect the expression of several genes selected in a pathway corresponding to the gene functions in the literature, and thus is limited to study the activation of the whole pathway. Whereas the PCR array, as a commercially widely-used array, is able to provide high-throughput information of the most important genes in a certain signaling pathway, and thus is able to analyse the activation of pathways comprehensively (Fig. 8). Therefore, the PCR array provides a more comprehensive, more efficient and more reliable approach to validate important pathways.

## Conclusion

In this paper, the signaling pathway PCR array, for the first time, is introduced to validate the signaling pathways results of bioinformatics analysis for the biomics data. By investigating the gene expression profiles of the most important genes in the TGF-β signaling pathway and the regulation of actin cytoskeleton pathway of the HUVECs after cultured on the bare and the TiN-coated NiTi alloys and the chitosan film for 4 h and 24 h, these two pathways were verified to be activated in the process of cell adhesion and cell growth on the NiTi alloys. In addition, these two pathways showed stronger activation in the 24 h-culturing groups versus the 4 h groups and became more favorable for cell adhesion and growth over time on the surface of the alloys. This study also provides a novel approach to perfect our research strategy and complete our technical roadmap for studying signaling pathways in the interaction between biomaterials and cells.

## Funding

National Natural Science Foundation of China (31271012), 973 Project (No. 2009CB930000), and the Natural Science Foundation of Jiangsu Province (BK20150599).

## Supplementary data

Supplementary data are available at *REGBIO* online.

*Conflict of interest statement*. None declared.

## Supplementary Material

Supporting Information
