# Supplementary material for: A high-throughput study on endothelial cell adhesion and growth mediated by adsorbed serum protein via signaling pathway PCR array
Source: Regen Biomater. 2017 Dec 13;5(1):25–34. doi: 10.1093/rb/rbx030 (PMC5798144; doi:10.1093/rb/rbx030)
Supplement: Supporting Information [file supporting_information_rbx030.docx]

**Supporting Information**

**Title:** A high-throughput study for endothelial cell adhesion and growth mediated by adsorbed serum protein via signaling pathway PCR array

**Author:** Xiaoying Lü, Yayun Qu, Ying Hong, Yan Huang, Yiwen Zhang, Fudan Zhang, Dayun Yang, Tingfei Xi, Deyuan Zhang

**Supplementary Table 1.** 30 significantly differentially expressed genes in bare NiTi-4h group analyzed by TGF-β/BMP signaling pathway PCR array (*p*<0.05).

**Supplementary Table 2.** 27 significantly differentially expressed genes in bare NiTi-24h group analyzed by TGF-β/BMP signaling pathway PCR array (*p*<0.05).

**Supplementary Table 3.** 38 significantly differentially expressed genes in TiN-coated NiTi-4h group analyzed by TGF-β/BMP signaling pathway PCR array (*p*<0.05).

**Supplementary Table 4.** 31 significantly differentially expressed genes in TiN-coated NiTi-24h group analyzed by TGF-β/BMP signaling pathway PCR array (*p*<0.05).

**Supplementary Table 5.** 30 significantly differentially expressed genes in bare NiTi-4h group analyzed by cytoskeleton regulators PCR array (*p*<0.05).

**Supplementary Table 6.** 24 significantly differentially expressed genes in bare NiTi-24h group analyzed by cytoskeleton regulators PCR array (*p*<0.05).

**Supplementary Table 7.** 29 significantly differentially expressed genes in bare NiTi-24h group analyzed by cytoskeleton regulators PCR array (*p*<0.05).

**Supplementary Table 8.** 29 significantly differentially expressed genes in bare NiTi-24h group analyzed by cytoskeleton regulators PCR array (*p*<0.05).

**Supplementary Table 1.** 30 significantly differentially expressed genes in bare NiTi-4h group analyzed by TGF-β/BMP signaling pathway PCR array (*p*<0.05).

| No. | Gene Symbol | p value | Fold Change |
| --- | --- | --- | --- |
| 1 | TGFB1 | 4.60E-06 | 12.26 |
| 2 | PDGFB | 1.60E-05 | 12.15 |
| 3 | ENG | 1.46E-07 | 11.68 |
| 4 | SMAD7 | 3.19E-05 | 8.97 |
| 5 | TGFB1I1 | 9.00E-06 | 8.66 |
| 6 | CHRD | 7.30E-08 | 7.27 |
| 7 | BMP6 | 3.08E-06 | 6.75 |
| 8 | BMP1 | 1.04E-06 | 5.35 |
| 9 | KANK4 | 6.17E-06 | 4.90 |
| 10 | SMAD6 | 1.43E-05 | 4.60 |
| 11 | ACVRL1 | 1.51E-06 | 4.46 |
| 12 | NODAL | 1.50E-04 | 3.96 |
| 13 | ACTB | 6.33E-07 | 3.92 |
| 14 | IGFBP3 | 7.11E-04 | 3.86 |
| 15 | UBASH3B | 2.69E-03 | 3.71 |
| 16 | PMEPA1 | 1.34E-03 | 3.49 |
| 17 | SERPINE1 | 2.28E-06 | 3.23 |
| 18 | SMURF1 | 9.56E-06 | 3.18 |
| 19 | CDKN1A | 4.41E-05 | 3.17 |
| 20 | COL1A1 | 1.09E-03 | 2.67 |
| 21 | PLAU | 4.12E-04 | 2.55 |
| 22 | MYC | 9.36E-05 | 2.55 |
| 23 | BAMBI | 1.30E-03 | 2.30 |
| 24 | GDF2 | 2.60E-03 | 2.25 |
| 25 | GDF6 | 3.27E-02 | 2.11 |
| 26 | TGFBR2 | 8.29E-05 | 2.09 |
| 27 | ACVR1 | 8.56E-05 | 2.03 |
| 28 | DCN | 5.86E-04 | 0.49 |
| 29 | GDF3 | 3.33E-04 | 0.45 |
| 30 | INHA | 1.56E-04 | 0.12 |

**Supplementary Table 2.** 27 significantly differentially expressed genes in bare NiTi-24h group analyzed by TGF-β/BMP signaling pathway PCR array (*p*<0.05).

| No. | Gene Symbol | p value | Fold Change |
| --- | --- | --- | --- |
| 1 | TGFB1 | 3.11E-05 | 22.26 |
| 2 | TGFB1I1 | 1.61E-04 | 12.83 |
| 3 | ENG | 4.65E-06 | 8.92 |
| 4 | PDGFB | 1.48E-04 | 8.67 |
| 5 | SMAD7 | 1.57E-04 | 7.60 |
| 6 | ACTB | 9.84E-05 | 7.43 |
| 7 | NODAL | 1.67E-02 | 7.11 |
| 8 | BMP6 | 9.97E-05 | 6.42 |
| 9 | BMP1 | 1.40E-04 | 5.37 |
| 10 | ACVRL1 | 3.73E-04 | 4.17 |
| 11 | MYC | 3.35E-04 | 3.44 |
| 12 | COL1A2 | 2.23E-03 | 3.40 |
| 13 | NOG | 4.12E-02 | 3.04 |
| 14 | PMEPA1 | 1.42E-04 | 2.65 |
| 15 | SMURF1 | 3.46E-05 | 2.62 |
| 16 | UBASH3B | 6.61E-03 | 2.45 |
| 17 | SERPINE1 | 2.28E-04 | 2.43 |
| 18 | TGFB3 | 5.04E-03 | 2.41 |
| 19 | CDKN1A | 8.60E-03 | 2.08 |
| 20 | ACVR1 | 1.60E-03 | 2.03 |
| 21 | NOV | 8.36E-04 | 0.47 |
| 22 | ACVR2A | 2.76E-03 | 0.45 |
| 23 | HPRT1 | 3.39E-04 | 0.45 |
| 24 | B2M | 2.12E-04 | 0.44 |
| 25 | FAS | 2.00E-03 | 0.37 |
| 26 | IL6 | 1.40E-04 | 0.33 |
| 27 | IFRD1 | 2.97E-04 | 0.20 |

**Supplementary Table 3.** 38 significantly differentially expressed genes in TiN-coated NiTi-4h group analyzed by TGF-β/BMP signaling pathway PCR array (p<0.05).

| No. | Gene Symbol | p value | Fold Change |
| --- | --- | --- | --- |
| 1 | SMAD7 | 8.27E-05 | 13.41 |
| 2 | TGFB1 | 2.56E-05 | 12.17 |
| 3 | ENG | 2.15E-06 | 9.71 |
| 4 | KANK4 | 2.19E-04 | 9.01 |
| 5 | TGFB1I1 | 3.07E-04 | 5.78 |
| 6 | JUNB | 1.32E-03 | 5.51 |
| 7 | SMAD6 | 1.64E-03 | 4.72 |
| 8 | BMP6 | 1.17E-04 | 4.53 |
| 9 | ACTB | 3.04E-06 | 4.52 |
| 10 | ACVRL1 | 6.88E-06 | 4.45 |
| 11 | CDKN1A | 1.34E-04 | 4.31 |
| 12 | COL1A1 | 3.16E-05 | 4.30 |
| 13 | BMP1 | 6.31E-06 | 4.19 |
| 14 | IGFBP3 | 5.07E-04 | 4.02 |
| 15 | UBASH3B | 1.53E-03 | 3.08 |
| 16 | BAMBI | 1.14E-04 | 3.08 |
| 17 | FSTL3 | 4.12E-03 | 2.82 |
| 18 | SMURF1 | 7.38E-07 | 2.45 |
| 19 | ACVR1 | 1.62E-03 | 2.27 |
| 20 | PMEPA1 | 2.10E-03 | 2.07 |
| 21 | GSC | 2.69E-03 | 2.00 |
| 22 | IFRD1 | 7.22E-03 | 0.48 |
| 23 | SMAD1 | 1.94E-05 | 0.47 |
| 24 | HERPUD1 | 5.69E-04 | 0.46 |
| 25 | HPRT1 | 2.00E-05 | 0.46 |
| 26 | SMAD5 | 6.53E-05 | 0.44 |
| 27 | GDF3 | 2.23E-04 | 0.44 |
| 28 | FGF2 | 8.04E-03 | 0.43 |
| 29 | NODAL | 1.54E-03 | 0.42 |
| 30 | COL1A2 | 9.26E-04 | 0.41 |
| 31 | TNFSF10 | 5.20E-07 | 0.35 |
| 32 | TGFB3 | 1.31E-03 | 0.32 |
| 33 | STAT1 | 3.31E-04 | 0.31 |
| 34 | IL6 | 4.75E-05 | 0.26 |
| 35 | TGFBI | 1.97E-05 | 0.24 |
| 36 | INHA | 1.34E-04 | 0.14 |
| 37 | TGFB2 | 1.20E-03 | 0.13 |
| 38 | BMP4 | 6.83E-05 | 0.12 |

**Supplementary Table 4.** 31 significantly differentially expressed genes in TiN-coated NiTi-24h group analyzed by TGF-β/BMP signaling pathway PCR array (*p*<0.05).

| No. | Gene Symbol | p value | Fold Change |
| --- | --- | --- | --- |
| 1 | TGFB1 | 3.24E-05 | 23.50 |
| 2 | TGFB1I1 | 7.57E-06 | 13.90 |
| 3 | NODAL | 6.46E-03 | 13.50 |
| 4 | PDGFB | 9.99E-04 | 11.44 |
| 5 | LEFTY1 | 1.66E-02 | 9.24 |
| 6 | ENG | 2.22E-06 | 7.86 |
| 7 | ACTB | 1.87E-04 | 7.35 |
| 8 | PMEPA1 | 1.06E-03 | 6.98 |
| 9 | SMAD7 | 1.99E-04 | 6.89 |
| 10 | ACVRL1 | 1.30E-05 | 5.21 |
| 11 | BMP6 | 7.88E-05 | 5.05 |
| 12 | BMP1 | 2.60E-05 | 4.44 |
| 13 | COL1A1 | 3.87E-03 | 3.66 |
| 14 | MYC | 8.83E-07 | 2.76 |
| 15 | TGFB3 | 3.40E-04 | 2.50 |
| 16 | BAMBI | 5.21E-04 | 2.46 |
| 17 | COL1A2 | 3.77E-03 | 2.41 |
| 18 | SMURF1 | 5.68E-04 | 2.16 |
| 19 | THBS1 | 7.97E-04 | 2.12 |
| 20 | CDKN1A | 7.59E-04 | 2.11 |
| 21 | UBASH3B | 1.97E-03 | 2.07 |
| 22 | HPRT1 | 6.50E-04 | 0.50 |
| 23 | EMP1 | 3.13E-04 | 0.49 |
| 24 | SMAD5 | 6.65E-04 | 0.49 |
| 25 | BMPR1A | 1.05E-05 | 0.48 |
| 26 | STAT1 | 5.29E-05 | 0.47 |
| 27 | BMPR1B | 2.92E-04 | 0.36 |
| 28 | B2M | 5.25E-05 | 0.35 |
| 29 | STK38L | 1.34E-04 | 0.35 |
| 30 | INHA | 1.77E-02 | 0.28 |
| 31 | FAS | 9.19E-04 | 0.24 |

**Supplementary Table 5.** 30 significantly differentially expressed genes in bare NiTi-4h group analyzed by cytoskeleton regulators PCR array (*p*<0.05).

| No. | Gene Symbol | p value | Fold Change |
| --- | --- | --- | --- |
| 1 | PAK4 | 3.69E-04 | 11.12 |
| 2 | LLGL1 | 9.32E-05 | 7.29 |
| 3 | ARHGEF11 | 4.48E-04 | 6.36 |
| 4 | MAPK13 | 1.90E-04 | 5.57 |
| 5 | VASP | 3.88E-05 | 4.60 |
| 6 | LIMK1 | 1.64E-04 | 4.52 |
| 7 | ACTB | 2.33E-04 | 4.03 |
| 8 | MARK2 | 6.77E-04 | 4.01 |
| 9 | MYLK | 2.26E-04 | 3.32 |
| 10 | ARAP1 | 1.93E-04 | 3.31 |
| 11 | CDC42EP2 | 3.98E-05 | 3.09 |
| 12 | CASK | 3.14E-06 | 2.54 |
| 13 | GSN | 1.42E-05 | 2.51 |
| 14 | WASL | 2.80E-03 | 2.50 |
| 15 | MAP4 | 1.54E-04 | 2.46 |
| 16 | DIAPH1 | 9.68E-05 | 2.45 |
| 17 | CLIP2 | 4.67E-04 | 2.33 |
| 18 | ARPC1B | 1.59E-03 | 2.25 |
| 19 | CFL1 | 4.01E-03 | 2.02 |
| 20 | MYLK2 | 3.65E-03 | 2.00 |
| 21 | DSTN | 2.77E-03 | 0.50 |
| 22 | HPRT1 | 5.95E-05 | 0.49 |
| 23 | CDC42 | 2.01E-04 | 0.46 |
| 24 | ARPC2 | 6.98E-05 | 0.46 |
| 25 | CCNA1 | 8.93E-04 | 0.46 |
| 26 | PHLDB2 | 5.96E-04 | 0.45 |
| 27 | STMN1 | 3.73E-03 | 0.41 |
| 28 | PPP3CA | 3.32E-03 | 0.41 |
| 29 | NCK1 | 2.30E-04 | 0.37 |
| 30 | MAP3K11 | 2.44E-02 | 0.36 |

**Supplementary Table 6.** 24 significantly differentially expressed genes in bare NiTi-4h group analyzed by cytoskeleton regulators PCR array (*p*<0.05).

| No. | Gene Symbol | p value | Fold Change |
| --- | --- | --- | --- |
| 1 | PAK4 | 4.90E-06 | 17.20 |
| 2 | LIMK1 | 1.43E-04 | 11.42 |
| 3 | ARHGEF11 | 6.79E-05 | 8.75 |
| 4 | LLGL1 | 4.81E-04 | 7.15 |
| 5 | ACTB | 1.61E-05 | 5.88 |
| 6 | ARAP1 | 3.21E-04 | 5.77 |
| 7 | MARK2 | 4.85E-04 | 5.66 |
| 8 | CLIP2 | 4.16E-06 | 4.89 |
| 9 | CDC42EP2 | 5.99E-04 | 4.63 |
| 10 | MAP4 | 1.67E-04 | 4.15 |
| 11 | WAS | 5.50E-03 | 4.04 |
| 12 | MAP3K11 | 3.08E-02 | 3.55 |
| 13 | WASL | 3.00E-05 | 3.50 |
| 14 | ARHGAP6 | 1.59E-02 | 3.24 |
| 15 | DIAPH1 | 4.79E-04 | 3.21 |
| 16 | TIAM1 | 4.82E-04 | 2.80 |
| 17 | CFL1 | 4.59E-04 | 2.66 |
| 18 | ARPC1B | 4.93E-03 | 2.33 |
| 19 | AURKB | 3.81E-04 | 2.30 |
| 20 | CASK | 3.90E-05 | 2.25 |
| 21 | WASF1 | 7.09E-05 | 2.25 |
| 22 | MAPK13 | 2.25E-04 | 2.24 |
| 23 | GSN | 2.44E-03 | 2.05 |
| 24 | CDK5R1 | 5.63E-03 | 0.38 |

**Supplementary Table 7.** 29 significantly differentially expressed genes in TiN-coated NiTi-4h group analyzed by cytoskeleton regulators PCR array (*p*<0.05).

| No. | Gene Symbol | p value | Fold Change |
| --- | --- | --- | --- |
| 1 | NCK2 | 1.42E-04 | 9.73 |
| 2 | PAK4 | 9.38E-06 | 8.57 |
| 3 | LLGL1 | 1.59E-06 | 6.68 |
| 4 | LIMK1 | 2.87E-05 | 5.14 |
| 5 | ACTB | 2.68E-06 | 4.48 |
| 6 | MAPK13 | 9.37E-05 | 4.24 |
| 7 | MYLK | 2.86E-04 | 3.57 |
| 8 | VASP | 4.40E-03 | 3.44 |
| 9 | MAP4 | 1.04E-05 | 2.43 |
| 10 | GSN | 2.61E-03 | 2.19 |
| 11 | ARPC1B | 2.75E-05 | 2.10 |
| 12 | ARPC3 | 2.34E-04 | 0.49 |
| 13 | HPRT1 | 4.97E-05 | 0.46 |
| 14 | CLIP1 | 5.37E-04 | 0.43 |
| 15 | CLASP1 | 4.43E-04 | 0.42 |
| 16 | ARPC2 | 7.33E-06 | 0.40 |
| 17 | CTTN | 1.45E-03 | 0.38 |
| 18 | ARPC5 | 2.81E-04 | 0.37 |
| 19 | DSTN | 1.05E-03 | 0.35 |
| 20 | NCK1 | 2.52E-04 | 0.33 |
| 21 | PIKFYVE | 1.21E-04 | 0.32 |
| 22 | PPP1R12A | 9.94E-05 | 0.31 |
| 23 | CDC42 | 1.23E-04 | 0.30 |
| 24 | SSH2 | 1.81E-04 | 0.29 |
| 25 | IQGAP2 | 6.66E-05 | 0.26 |
| 26 | ACTR2 | 7.57E-04 | 0.23 |
| 27 | PHLDB2 | 6.53E-06 | 0.20 |
| 28 | PPP3CA | 8.68E-04 | 0.19 |
| 29 | CCNA1 | 1.01E-04 | 0.06 |

**Supplementary Table 8.** 29 significantly differentially expressed genes in TiN-coated NiTi-24h group analyzed by cytoskeleton regulators PCR array (*p*<0.05).

| No. | Gene Symbol | p value | Fold Change |
| --- | --- | --- | --- |
| 1 | PAK4 | 5.72E-04 | 21.81 |
| 2 | LIMK1 | 5.54E-05 | 14.21 |
| 3 | LLGL1 | 4.03E-04 | 9.43 |
| 4 | MAP3K11 | 2.54E-02 | 8.11 |
| 5 | ARHGEF11 | 6.14E-06 | 7.72 |
| 6 | MARK2 | 6.87E-04 | 7.53 |
| 7 | ARAP1 | 2.38E-04 | 7.13 |
| 8 | ACTB | 7.90E-05 | 6.54 |
| 9 | CLIP2 | 4.92E-04 | 5.81 |
| 10 | CDC42EP2 | 8.72E-05 | 5.50 |
| 11 | MAP4 | 2.78E-06 | 5.00 |
| 12 | DIAPH1 | 2.03E-06 | 3.51 |
| 13 | MAPK13 | 1.51E-03 | 3.36 |
| 14 | ARPC1B | 2.27E-05 | 3.28 |
| 15 | CFL1 | 6.41E-06 | 3.05 |
| 16 | ARHGAP6 | 5.50E-03 | 2.75 |
| 17 | GSN | 4.66E-05 | 2.36 |
| 18 | WASL | 2.67E-03 | 2.26 |
| 19 | AURKB | 1.03E-04 | 2.25 |
| 20 | TIAM1 | 8.13E-03 | 2.25 |
| 21 | LIMK2 | 4.13E-04 | 2.13 |
| 22 | PPP3CB | 1.96E-03 | 2.10 |
| 23 | EZR | 2.26E-03 | 2.08 |
| 24 | HPRT1 | 3.10E-05 | 0.50 |
| 25 | FNBP1L | 1.83E-03 | 0.48 |
| 26 | B2M | 9.79E-05 | 0.45 |
| 27 | CALD1 | 3.76E-05 | 0.45 |
| 28 | ACTR2 | 8.82E-04 | 0.44 |
| 29 | PHLDB2 | 2.46E-05 | 0.39 |
